# *Clostridium perfringens* Alpha-Toxin Induces Gm1a Clustering and Trka Phosphorylation in the Host Cell Membrane

**DOI:** 10.1371/journal.pone.0120497

**Published:** 2015-04-24

**Authors:** Teruhisa Takagishi, Masataka Oda, Michiko Kabura, Mie Kurosawa, Kaori Tominaga, Shiori Urano, Yoshibumi Ueda, Keiko Kobayashi, Toshihide Kobayashi, Jun Sakurai, Yutaka Terao, Masahiro Nagahama

**Affiliations:** 1 Department of Microbiology, Faculty of Pharmaceutical Sciences, Tokushima Bunri University, Yamashiro-cho 180, Tokushima, 770–8514, Japan; 2 Division of Microbiology and Infectious Diseases, Niigata University Graduate School of Medical and Dental Sciences, 2–5274, Gakkocho-dori, Chuo-ku, Niigata, 951–8514, Japan; 3 Hematology and Immunology, Kanazawa Medical University, 1–1 Daigaku, Uchinada-cho, Kahoku-gun, Ishikawa, 920–0293, Japan; 4 Lipid Biology Laboratory, Rikagaku Kenkyujo (RIKEN), 2–1 Hirosawa, Wako, Saitama, 351–0198, Japan; Institute Pasteur, FRANCE

## Abstract

*Clostridium perfringens* alpha-toxin elicits various immune responses such as the release of cytokines, chemokines, and superoxide via the GM1a/TrkA complex. Alpha-toxin possesses phospholipase C (PLC) hydrolytic activity that contributes to signal transduction in the pathogenesis of gas gangrene. Little is known about the relationship between lipid metabolism and TrkA activation by alpha-toxin. Using live-cell fluorescence microscopy, we monitored transbilayer movement of diacylglycerol (DAG) with the yellow fluorescent protein-tagged C1AB domain of protein kinase C-γ (EYFP-C1AB). DAG accumulated at the marginal region of the plasma membrane in alpha toxin-treated A549 cells, which also exhibited GM1a clustering and TrkA phosphorylation. Annexin V binding assays showed that alpha-toxin induced the exposure of phosphatidylserine on the outer leaflet of the plasma membrane. However, H148G, a variant toxin which binds cell membrane and has no enzymatic activity, did not induce DAG translocation, GM1a clustering, or TrkA phosphorylation. Alpha-toxin also specifically activated endogenous phospholipase Cγ-1 (PLCγ-1), a TrkA adaptor protein, via phosphorylation. U73122, an endogenous PLC inhibitor, and siRNA for PLCγ-1 inhibited the formation of DAG and release of IL-8. GM1a accumulation and TrkA phosphorylation in A549 cells treated with alpha-toxin were also inhibited by U73122. These results suggest that the flip-flop motion of hydrophobic lipids such as DAG leads to the accumulation of GM1a and TrkA. We conclude that the formation of DAG by alpha-toxin itself (first step) and activation of endogenous PLCγ-1 (second step) leads to alterations in membrane dynamics, followed by strong phosphorylation of TrkA.

## Introduction

The anaerobic, spore-forming bacterium *Clostridium perfringens* is a ubiquitous and widely distributed pathogen in nature [1, 2]. It is found in soil, sewage, and the gastrointestinal tracts of humans and many animals [1, 3–5]. *C*. *perfringens*-induced myonecrosis or gas gangrene, is a major invasive and fulminant gram-positive infection in humans [6], characterized by rapid destruction of viable, healthy tissue [7]. Shock and organ failure frequently accompany gas gangrene and mortality exceeds 50% when patients become bacteremic [6]. Radical amputation remains the single best life-saving treatment for *C*. *perfringens* infection [7]. A characteristic feature of infection with *C*. *perfringens* is an absence of neutrophils within the area of necrosis associated with *C*. *perfringens* growth and the accumulation of neutrophils at the border of the necrotic region [8, 9].

*C*. *perfringens* alpha-toxin, which possesses lethal hemolytic and dermonecrotic activities, is the major virulence factor associated with the pathogenesis of gas gangrene. Bacterial phospholipase Cs (PLCs) hydrolyze phosphatidylcholine (PC) and sphingomyelin (SM) and are important virulence factors in *Listeria monocytogenes*, *Pseudomonas aeruginosa*, and several *Clostridium* spp. [10–12]. Alpha-toxin exhibits PLC and sphingomyelinase activities, is highly cytotoxic and myotoxic, and can promote hemolysis and the release of superoxide radicals and inflammatory cytokines [13–19]. Bunting et al. reported that alpha-toxin induces vascular permeability, myocardial dysfunction, and localized neutrophil accumulation [9]. Interleukin-8 (IL-8), which belongs to the CXC chemokine family, is a pivotal chemotactic cytokine responsible for the recruitment of neutrophils and macrophages [20, 21]. Therefore, it is possible that the unique behavior of neutrophils in gas gangrene is dependent on IL-8 released from macrophages and/or endothelial cells that have been activated by the *C*. *perfringens* toxin.

Alpha-toxin simultaneously induces formation of diacylglycerol (DAG) by activating endogenous PLC and phosphorylation of ERK1/2, NFκB, and p38 MAPK by activating tyrosine kinase A (TrkA) in human lung adenocarcinoma epithelial (A549) cells, leading to the release of IL-8 [22]. The Trp-84 and Tyr-85 residues of alpha-toxin specifically interact with ganglioside GM1a to activate TrkA in A549 cells [23]. Alpha-toxin activates protein kinase C (PKC) and the MEK/ERK-NFκB pathways in ganglioside-deficient cells, leading to the production of reactive oxygen species and cell death [24]. In addition, alpha-toxin internalization in the cytosol of Don Q cells, a ganglioside-deficient cell line, activates ERK1/2 [24].

DAG is a small, simple lipid with a key role in lipid metabolism and lipid-mediated signaling [25–27]. Extensive studies have demonstrated the importance of DAG as a second messenger that activates PKC through direct binding to the C1 domain, resulting in PKC translocation to the membrane [28, 29]. The flip-flop of DAG in the plasma membrane influences membrane dynamics by stimulating flipping of other molecules [30, 31]. Alpha-toxin induces DAG formation by activating endogenous PLC and the phosphorylation of phosphatidylinositol 3-kinase and 3-phosphoinositide-dependent kinase 1 by activating TrkA in rabbit neutrophils, ultimately resulting in the generation of superoxide through ERK1/2-associated signaling events [13].

We investigated the relationship between DAG formation and TrkA activation in response to alpha-toxin. We also characterized the roles of alpha-toxin and endogenous PLCγ-1 in phospholipid metabolism, TrkA activation, and subsequent IL-8 release.

## Materials and Methods

### Materials

The PLC inhibitor U73122 and its inactive analogue U73343 were purchased from Calbiochem (San Diego, CA). Antibodies against rabbit anti-phospho-TrkA (Tyr490) (catalog #9141), anti-phospho-PLCγ-1 (Ser1248) (catalog #4510), anti-phospho-PLCγ-2 (Tyr759) (catalog #3874), anti-phospho-PLCβ-3 (Ser537) (catalog #2481), anti- PLCγ-1 (catalog #2822), and anti-β-actin (catalog #4967) were obtained from Cell Signaling Technology (Danvers, MA). BODIPY FL C_5_-ganglioside GM1a and Alexa Fluor 546-conjugated goat anti-rabbit IgG (catalog #A-11010) were purchased from Molecular Probes (Eugene, OR). Horseradish peroxidase-labeled goat anti-rabbit IgG (catalog #7074) and an ECL Western blotting kit were obtained from GE Healthcare (Tokyo, Japan). Hoechst 33342 was obtained from Dojindo Laboratories (Kumamoto, Japan). All other drugs were of analytical grade.

### Cell Culture

Human lung adenocarcinoma epithelial (A549) cells were obtained from Riken Cell Bank (Tsukuba, Japan). Cells were cultured at 37°C in DMEM supplemented with 10% fetal bovine serum (FBS, AusGeneX, Oxenford, AU), 100 units/mL penicillin, and 100 μg/mL streptomycin.

### Purification of wild-type and variant alpha-toxin

Recombinant forms of pHY300PLK harboring the structural genes of wild-type or H148G variant alpha-toxin [32] were introduced into *Bacillus subtilis* ISW1214 by transformation. Transformants were grown in Luria-Bertani broth containing 50 μg ampicillin/mL to an optical density at 600 nm of 0.8 to 0.85, with continuous aeration. Purification of wild-type or H148G variant alpha-toxin was performed as described [32] and yielded products of greater than 98% purity.

### Confocal detection of diacylglycerol

The expression vector for the yellow fluorescent protein-tagged C1AB domain of protein kinase C-γ (EYFP-C1AB) was prepared as described [33]. The EYFP-C1AB plasmid was transfected into A549 cells using the Neon Transfection System (Life Technologies Japan, Tokyo, Japan) according to manufacturer instructions. After electroporation, cells were transferred to poly-L-lysine glass-bottom dishes (MatTek, Ashland, MA) containing pre-warmed antibiotic-free media and incubated at 37°C. After 24 h, the cells were incubated with 1.0 μg/mL wild-type or H148G alpha-toxin at 37°C and visualized with a Nikon A1 scanning-laser confocal microscope (Tokyo, Japan).

### Cell staining and imaging

For immunofluorescence labeling, A549 cells (1.0 × 10^5^ cells/mL) were seeded on poly-L-lysine glass-bottom dishes (MatTek). After incubation, cells were treated with alpha-toxin (1.0 μg/mL) at 37°C for 60 min, fixed with 4% paraformaldehyde in PBS at room temperature for 15 min, and then washed three times with PBS. Fixed cells were permeabilized with PBS containing 0.1% Triton X-100 for 5 min, and then treated with PBS containing 4% BSA for 30 min. The cells were incubated with primary antibody for 1 h followed by the appropriate secondary antibody at room temperature for 1 h. Nuclei were stained with Hoechst 33342. Stained cells were visualized using BIOREVO (BZ-9000, Keyence, Osaka, Japan) with the associated analysis software package (BZ-H2A). Fluorescence intensity was measured by using the Dynamic Cell Count application of BIOREVO. Values were calculated as the average of three fields. Labeling with annexin V was performed by adding Alexa Fluor 488-conjugated annexin V with wild-type or H148G alpha-toxin in solution. Stained cells were visualized by confocal microscopy.

### Clustering of GM1a in A549 cells

A549 cells were seeded on poly-L-lysine glass-bottom dishes (MatTek), 500 nM BODIPY-GM1a was added, and the cells were incubated at 4°C for 15 min. The cells were then treated with wild-type or H148G alpha-toxin (1.0 μg/mL) at 37°C, fixed with 4% paraformaldehyde in PBS at room temperature for 15 min, and washed three times with PBS. The nuclei were stained with Hoechst 33342. Stained cells were visualized on the BIOREVO and fluorescence intensity was measured as described above. Values were calculated as the average of three fields.

### Determination of diacylglycerol

A549 cells (1 × 10^7^ cells/mL) were pretreated with various amounts of U73122 or U73343 at 37°C for 60 min, and then incubated with or without alpha-toxin (1.0 μg/mL) at 37°C for 60 min. Intracellular diacylglycerol levels were determined as described previously [34].

### IL-8 ELISA

Immunoreactive IL-8 was quantified in cell culture supernatants with a double-antibody ELISA kit using rIL-8 as a standard (R&D Systems, Minneapolis, MN, USA).

### Phosphorylation of PLC isoforms induced by alpha-toxin

A549 cells were incubated with alpha-toxin (1.0 μg/mL) at 37°C in culture medium. The reaction was terminated by adding 0.5 mL ice-cold 10% trichloroacetic acid containing 0.1 mM Na_3_VO_4_ and incubating on ice for 30 min. The precipitate was collected by centrifugation at 15,000 × *g* for 15 min. Phosphorylated proteins were electrophoresed by SDS-PAGE and transferred to Immobilon polyvinylidene difluoride membranes (Millipore). The membranes were incubated with 5% (wt/vol) nonfat milk in TBST buffer (10 mM Tris-HCl, pH 8.0, 150 mM NaCl, 0.05% [vol/vol] Tween 20) and probed with specific rabbit monoclonal antibodies against various phosphorylated and unphosphorylated proteins (diluted 1:1,000 in TBST buffer). Detection was conducted using the enhanced chemiluminescence kit. Quantitative analysis was performed by densitometry (LAS-4000, Fujifilm, Japan).

### Transfection with siRNA for PLCγ-1

Small interfering RNAs (siRNA) targeting PLCγ-1 and a siRNA negative control were obtained from Qiagen (Valencia, CA, USA). A549 cells were mixed with 10 nM siRNA for PLCγ-1 or negative control siRNA (NC-siRNA) and then electroporated using the Neon Transfection System (Life Technologies Japan). After electroporation, cells were transferred to 24-well plates or a poly-L-lysine glass-bottom dish containing pre-warmed antibiotic-free media and incubated at 37°C. Experiments were performed 48 h after transfection. Silencing was verified by western blotting with anti-PLCγ-1 antibody (Cell Signaling Technology).

### Statistical Analysis

All data presented are expressed as the mean ± S.E of at least three replicates. Mean values were compared by using the Student’s *t*-test and *p* < 0.01 was considered statistically significant.

## Results

### DAG accumulation in the plasma membrane

**Fig 1:**
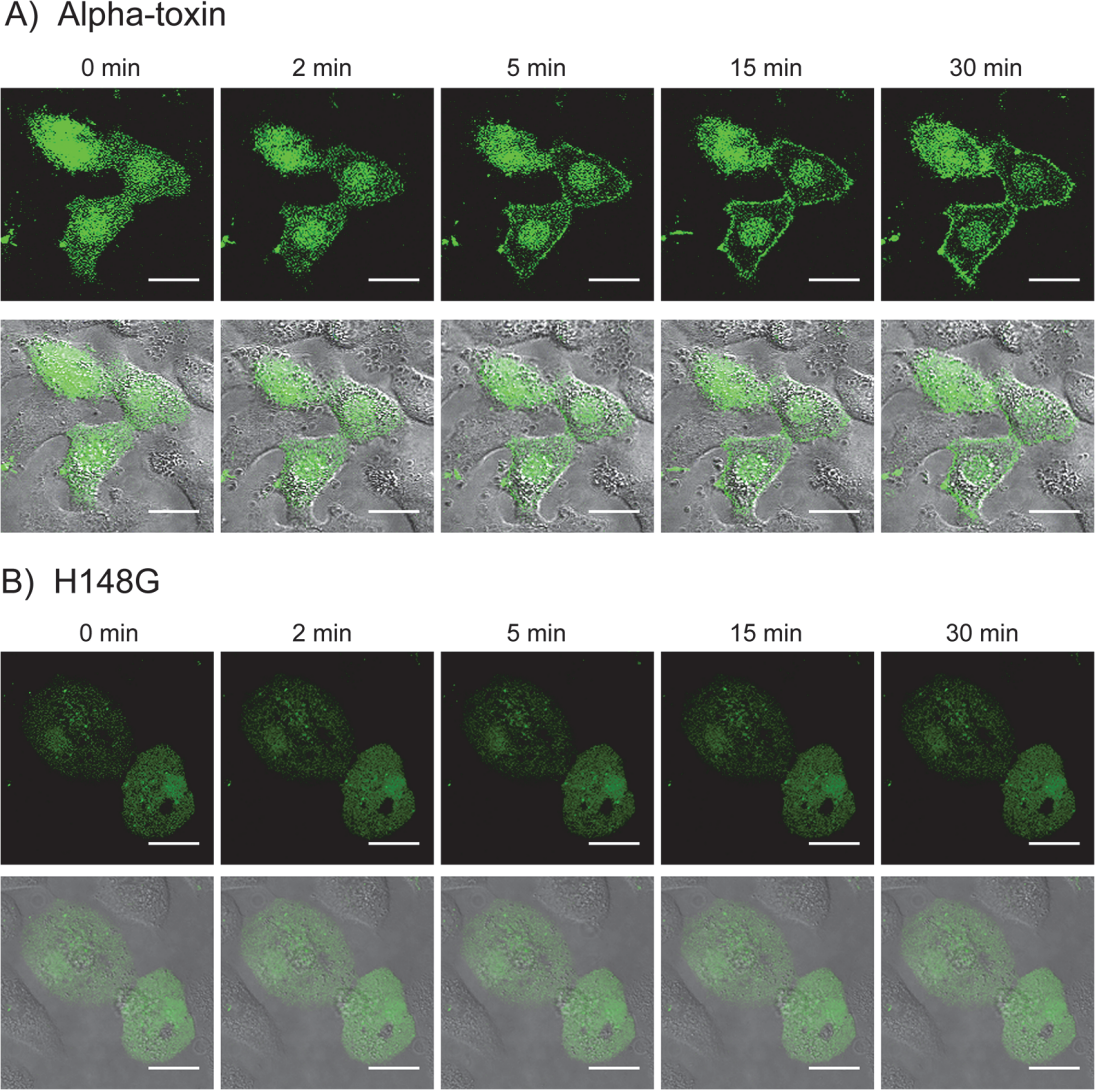
Transbilayer movement of DAG on the membrane treated with alpha-toxin. DNA transfection was used to express EYFP-C1AB in A549 cells. After 24 h, cells were incubated with 1.0 μg/mL wild-type alpha-toxin (A) or H148G alpha-toxin (B) at 37°C. The cells were visualized by confocal fluorescence microscopy. Scale bar, 10 μm.

To detect DAG in the plasma membrane of A549 cells, we used an EYFP-C1AB probe, an enhanced yellow fluorescent protein (EYFP) fused to the C1A and C1B domain of PLC-γ that is capable of binding DAG and phorbol ester [35]. Transient transfection of EYFP-C1AB into A549 cells yielded homogenous fluorescence across the cytosol and nucleus (Fig 1A). Addition of alpha-toxin led to translocation of EYFP-C1AB from the cytosol to the marginal region of the plasma membrane within minutes. DAG translocation was not observed when H148G, an inactive variant [14], was used (Fig 1B).

**Fig 2:**
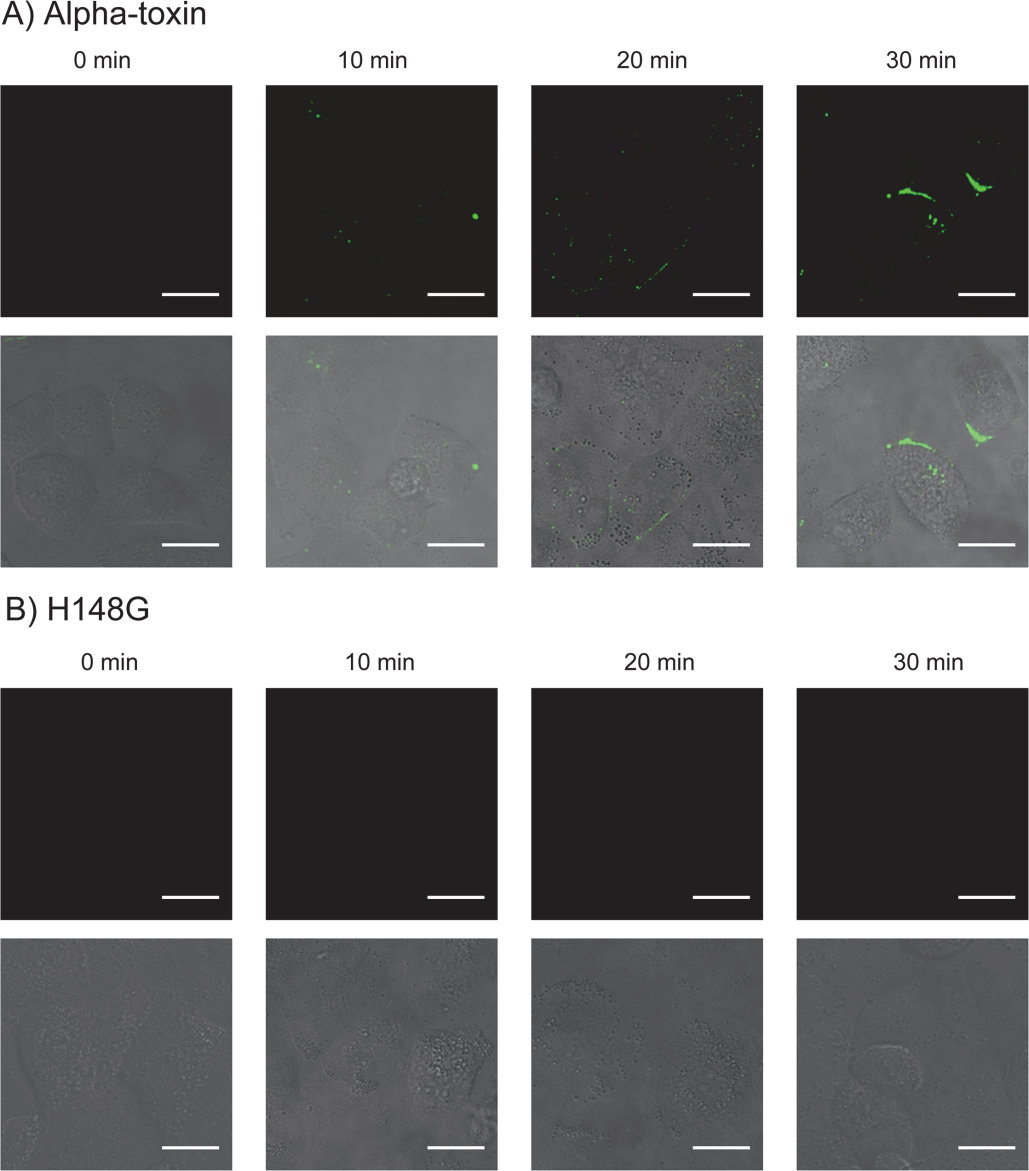
Alpha-toxin induced flip-flop motion in A549 cells. A549 cells were treated with 1.0 μg/mL wild-type alpha-toxin (A) or H148G alpha-toxin (B) in the presence of Alexa Fluor 488-conjugated annexin V. Fluorescence and differential interference contrast images were acquired at 0, 10, 20, and 30 min after the addition of alpha-toxin. Scale bar, 10 μm.

Next, to evaluate alpha-toxin toxicity in A549 cells, we measured the exposure of phosphatidylserine on the outer leaflet of the plasma membrane [36] by binding an Alexa Fluor 488 annexin V conjugate. Significant labeling with annexin V was observed 5 min after incubation with wild-type alpha-toxin (Fig 2A), but not with the H148G variant (Fig 2B). Ueda et al. reported that DAG formed in MDCK cells treated with alpha-toxin was transferred from the outer leaflet to the inner leaflet of the cell membrane [33]. This suggests alpha-toxin induced phosphatidylserine exposure via flip-flop of DAG.

### GM1a clustering and TrkA phosphorylation at the plasma membrane

**Fig 3:**
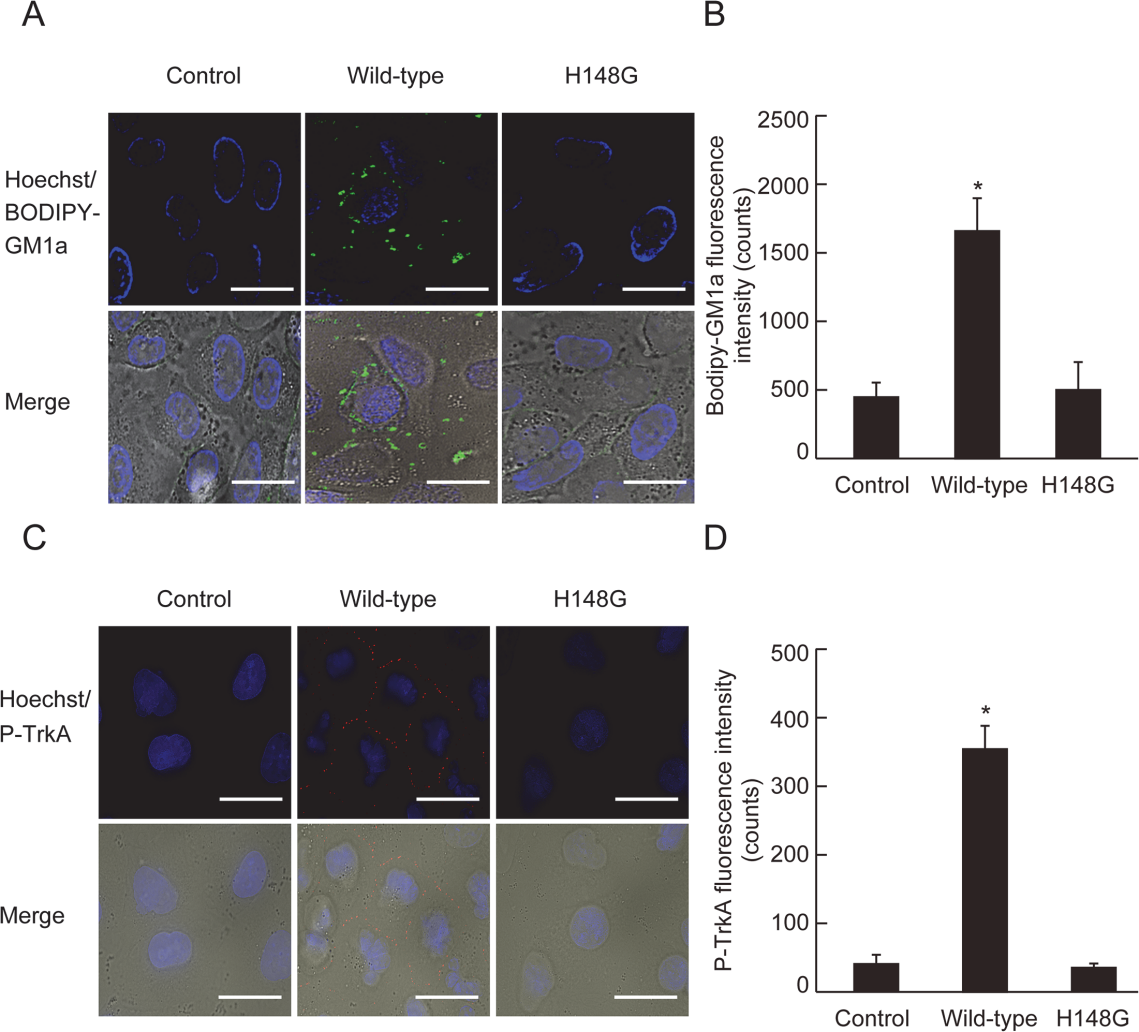
Clustering of GM1a and phosphorylation of TrkA in the membrane of cells treated with alpha-toxin. (A) A549 cells stained with BODIPY-GM1a were incubated with 1.0 μg/mL wild-type alpha-toxin or H148G alpha-toxin at 37°C for 60 min. The cells were fixed in 4% paraformaldehyde and stained with Hoechst 33342. GM1a (green) and nuclei (blue) were visualized by fluorescence microscopy. Scale bar, 10 μm. (B) Bodipy-GM1a fluorescence intensity was measured as described in Materials and Methods. Values represent the mean ± SE; n = 3; *, *p* < 0.01. (C) A549 cells were incubated with 1.0 μg/mL wild-type or H148G alpha-toxin at 37°C for 60 min. The cells were fixed, permeabilized, and stained with phospho-TrkA antibody and Hoechst 33342. Phospho-TrkA (red) and nuclei (blue) were visualized by fluorescence microscopy. Scale bar, 10 μm. (D) Phospho-TrkA fluorescence intensity was measured as described in Materials and Methods. Values represent the mean ± SE; n = 5; *, *p* < 0.01.

Next, we investigated the localization of GM1a and phosphorylated TrkA. When the BODIPY-GM1a-labeled cells were incubated with alpha-toxin (1.0 μg/mL), fluorescence was localized to the marginal region of the plasma membrane (Fig 3A). TrkA phosphorylation was also detected at the marginal region of the plasma membrane in cells treated with alpha-toxin (Fig 3C) but not the inactive variant (Fig 3A and 3C), thus indicating that active alpha-toxin was responsible for the clustering of GM1a and TrkA phosphorylation.

### Endogenous PLC contributes to the clustering of GM1a and the phosphorylation of TrkA

**Fig 4:**
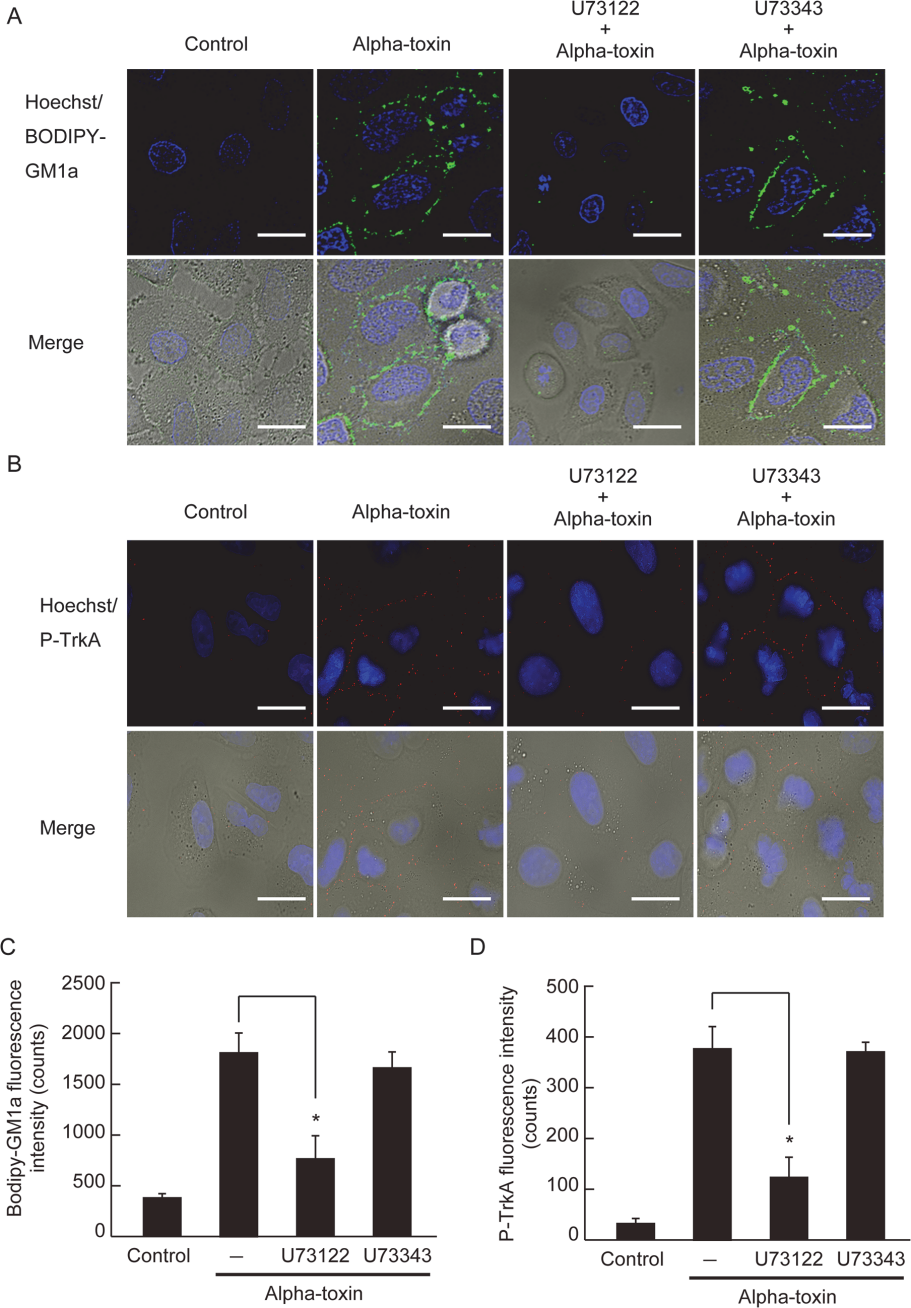
Inhibition of endogenous PLC affected the clustering of GM1a and phosphorylation of TrkA. (A) A549 cells were preincubated with 40 μM U73122 (endogenous PLC inhibitor) or U73343 (U73122 analogue) at 37°C for 60 min. The treated cells were stained with BODIPY-GM1a and incubated with 1.0 μg/mL wild-type or H148G alpha-toxin at 37°C for 60 min. The cells were fixed in 4% paraformaldehyde and stained with Hoechst 33342. GM1a (green) and nuclei (blue) were visualized by fluorescence microscopy. Scale bar, 10 μm. (B) A549 cells were preincubated with 40 μM U73122 or U73343 at 37°C for 60 min. The treated cells were incubated with 1.0 μg/mL wild-type or H148G alpha-toxin at 37°C for 60 min. The cells were fixed, permeabilized, and stained with phospho-TrkA antibody and Hoechst 33342. Phospho-TrkA (red) images and nuclei (blue) were visualized by fluorescence microscopy. Scale bar, 10 μm. (C) Bodipy-GM1a fluorescence intensity was measured as described in Materials and Methods. Values represent the mean ± SE; n = 3; *, *p* < 0.01. (D) Phospho-TrkA fluorescence intensity was measured as described in Materials and Methods. Values represent the mean ± SE; n = 5; *, *p* < 0.01.

**Fig 5:**
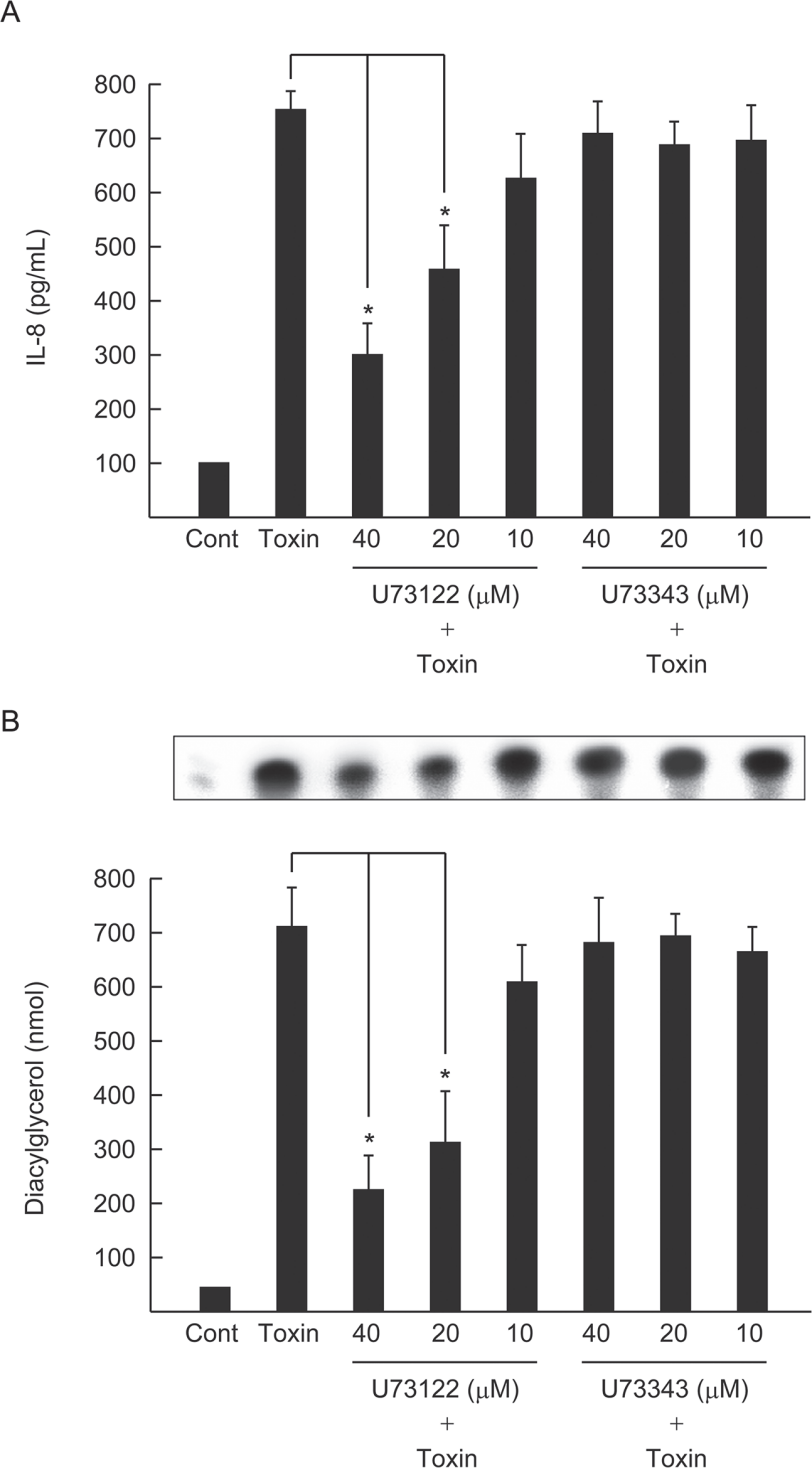
Inhibition of endogenous PLC prevented the alpha-toxin-induced release of IL-8 and formation of diacylglycerol. (A) A549 cells were pretreated with various amounts of U73122 or U73343 at 37°C for 60 min, and then incubated with or without alpha-toxin (1.0 μg/mL) at 37°C for 3 h. The concentration of IL-8 in culture supernatants was determined by ELISA. (B) A549 cells were pretreated with various amounts of U73122 or U73343 at 37°C for 60 min, and then incubated with or without alpha-toxin (1.0 μg/mL) at 37°C for 60 min and intracellular DAG levels were determined. Values represent mean ± S.E.; *n* = 4; *, *p* < 0.01.

Endogenous PLC plays an important role in the pathogenic activities induced by alpha-toxin such as hemolysis and superoxide generation [13, 37]. We examined the effect of an inhibitor of endogenous PLC (U73122) on GM1a clustering and TrkA phosphorylation in cells treated with alpha-toxin. Cells were preincubated with 40 μM U73122, followed by incubation with alpha-toxin. Treatment with U73122 inhibited alpha-toxin-induced GM1a accumulation and TrkA phosphorylation, but U73343, an inactive analog of U73122, did not elicit this effect (Fig 4A and 4B). In addition, U73122 inhibited IL-8 release and DAG formation in a dose-dependent manner, while U73343 did not (Fig 5A and 5B). This indicates that the activity of endogenous PLC is required for alpha-toxin-induced clustering of GM1a and TrkA phosphorylation, which could play a role in pathogenesis.

### Alpha-toxin specifically activates PLCγ-1

**Fig 6:**
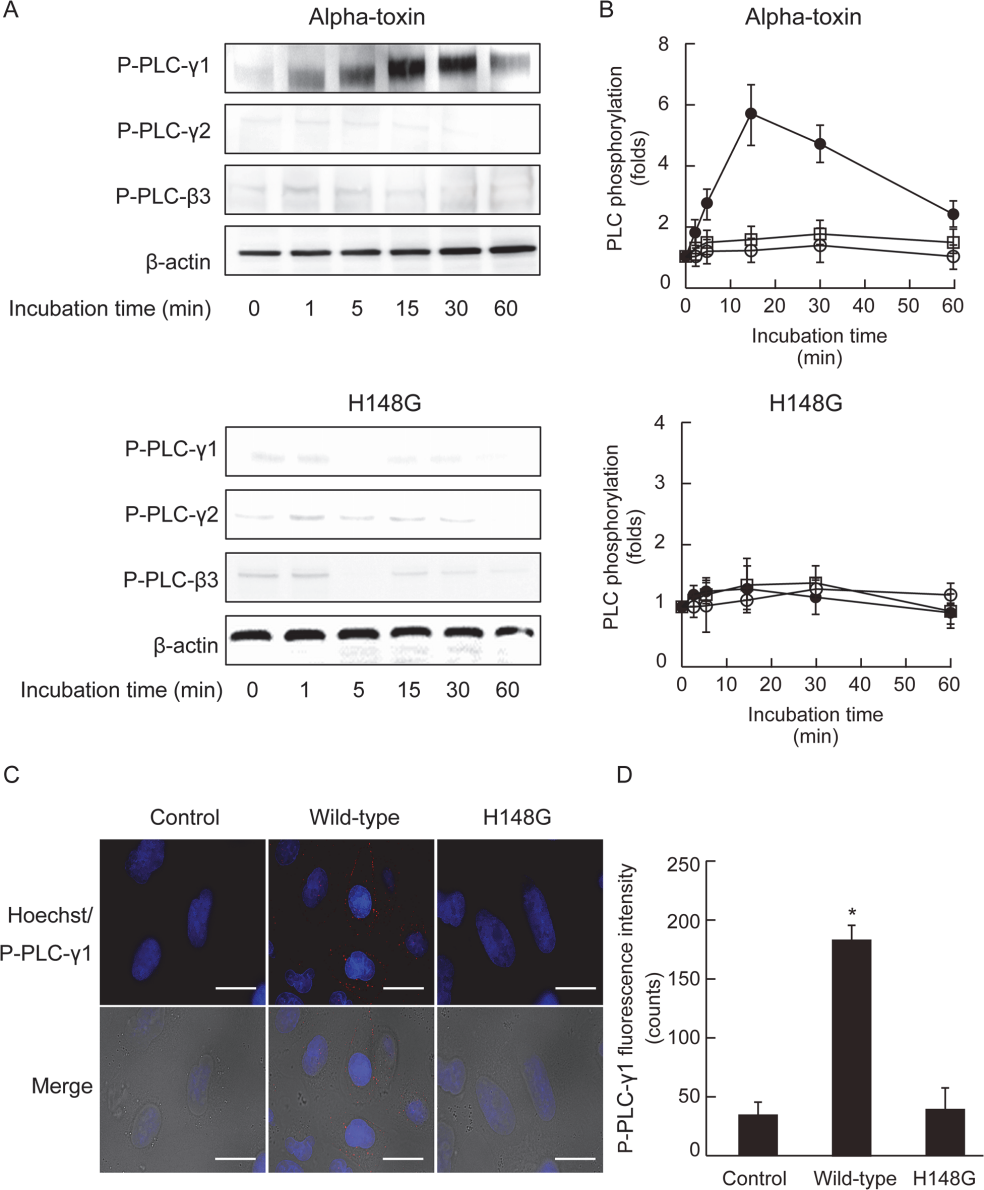
Alpha-toxin stimulated the phosphorylation of phospholipase Cγ-1. (A) A549 cells were incubated with 1.0 μg/mL wild-type alpha-toxin or 1.0 μg/mL H148G alpha-toxin at 37°C. Cell lysates were separated by SDS-PAGE and blotted with antibodies to phospho-PLCγ-1, phospho-PLCγ-2, and phospho-PLCβ-3. (B) Phosphorylated of PLCγ-1 (black circles), PLCγ-2 (white circles), and PLCβ-3 (white squares) in untreated cells was set to 1. Values represent the mean ± SE; n = 5. (C) A549 cells were incubated with 1.0 μg/mL wild-type or H148G alpha-toxin at 37°C for 60 min. The cells were fixed, permeabilized, and stained with phospho-PLCγ-1 antibody and Hoechst 33342. Phospho-PLCγ-1 (red) and nuclei (blue) were visualized by fluorescence microscopy. Scale bar, 10 μm. (D) Phospho- PLCγ-1 fluorescence intensity was measured as described in Materials and Methods. Values represent the mean ± SE; n = 5; *, *p* < 0.01.

Obermeier et al. reported that PLCγ-1 interacts with Tyr-785 of TrkA [38, 39]. Upon exposure to alpha-toxin, PLCγ-1 phosphorylation reached a maximum within 15 min and then decreased over time; however, the toxin had no effect on the phosphorylation of PLCγ-2 or PLCβ-3 (Fig 6A). PLCγ-1 phosphorylation was detected at the margins of the plasma membrane in cells treated with alpha-toxin (Fig 6C). In contrast, the inactive variant of alpha-toxin was unable to induce PLCγ-1 phosphorylation (Fig 6A and 6C).

**Fig 7:**
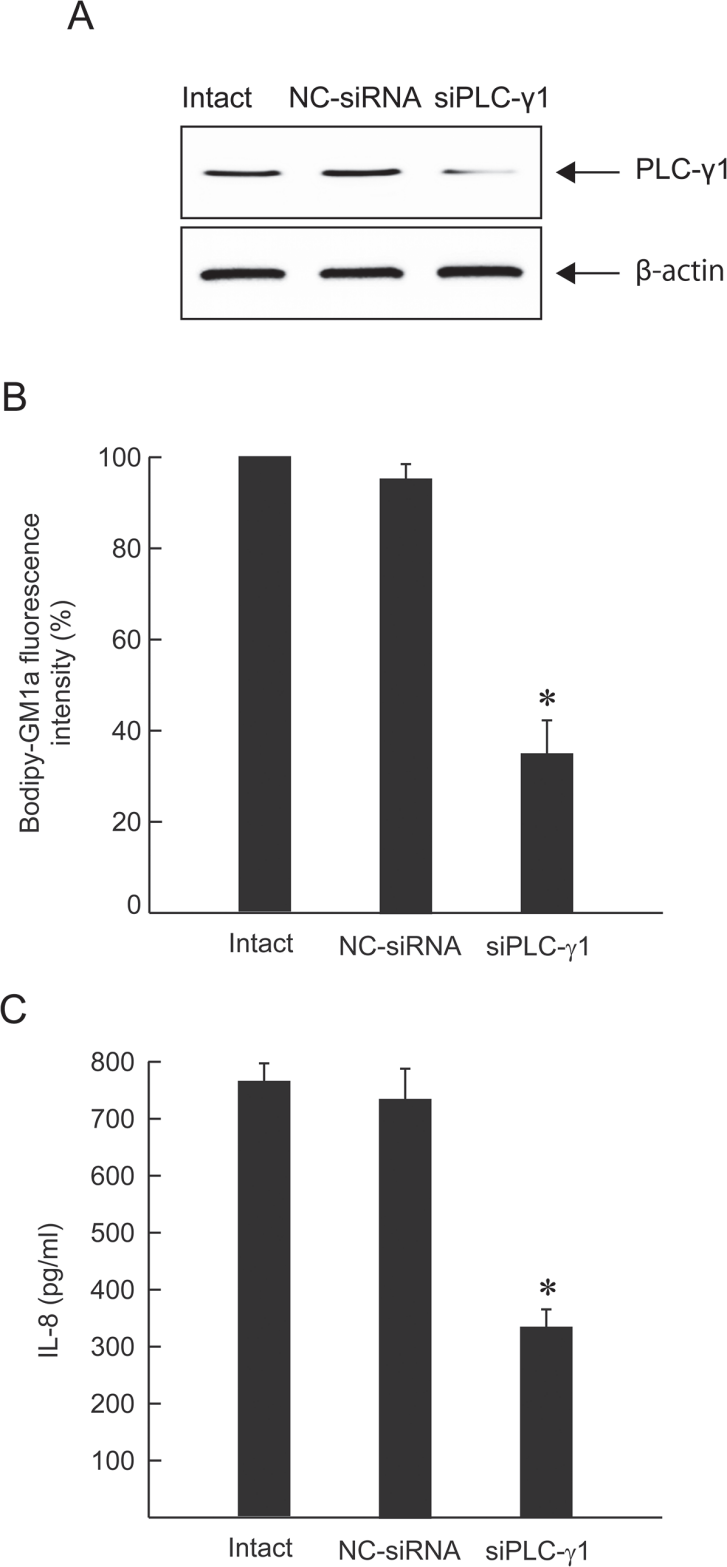
Effect of siRNA on clustering of GM1a and release of IL-8. A549 cells were transfected with siPLCγ-1 or NC-siRNA (10 nM). (A) Expression of PLCγ-1 was detected by western blotting with anti-PLCγ-1 and anti-β-actin antibodies. (B) Intact cells, NC-siRNA-treated cells, or siPLCγ-1-treated cells were stained with BODIPY-GM1a and incubated with 1.0 μg/mL alpha-toxin at 37°C for 60 min. The cells were fixed in 4% paraformaldehyde and analyzed by fluorescence microscopy. Fluorescence intensity was measured. The clustering of GM1a in the intact cells was set as the maximal response (100%) against which all other results were compared. Values represent the mean ± SE; n = 3; *, *p* < 0.01. (C) siRNA-treated cells were incubated with 1.0 μg/mL alpha-toxin at 37°C for 3 h. The concentration of IL-8 in the culture supernatants was determined by ELISA. Values represent the mean ± SE; n = 5; *, *p* < 0.01.

Transfection of A549 cells with PLCγ-1-specific siRNA significantly reduced PLCγ-1 protein expression, whereas a negative control siRNA (NC-siRNA) had no effect (Fig 7A). PLCγ-1 knockdown cells displayed a significant reduction in the fluorescence intensity of GM1a and release of IL-8 induced by alpha-toxin (Fig 7B and 7C). These results indicated that PLCγ-1 contributes to the pathogenic activity of alpha-toxin.

## Discussion

In this study, we demonstrated that the release of IL-8 induced by alpha-toxin depends on DAG localization at the cell membrane through the metabolism of PC. This activity was induced by the enzymatic activity of the bacterial toxin and the activation of endogenous PLCγ-1 via TrkA. Alpha-toxin induced translocation of a DAG-binding probe (EYFP-C1AB) at the marginal region of the plasma membrane, but an inactive toxin did not elicit this effect, indicating that the enzymatic activity of alpha-toxin itself plays a role in the formation of DAG. Annexin V-labeled cells were detected after 10 min incubation with alpha-toxin, suggesting that the toxin induced the exposure of phosphatidylserine via the flip-flop of DAG. In addition, the localization of GM1a and the phosphorylation of TrkA at the marginal region of the plasma membrane were induced by alpha-toxin, but not by H148G. The flip-flop of DAG in the plasma membrane influences membrane dynamics [18, 19]. Furthermore, Ichikawa et al. reported that GM1a clustering promotes TrkA enrichment in lipid rafts and the subsequent activation of downstream signal transduction pathways [40]. Therefore, DAG-induced transbilayer motion leads to the interaction of GM1a and TrkA at the plasma membrane.

Alpha-toxin induced the formation of DAG using PC, phosphatidylethanolamine, and to a lesser extent, phosphatidylinositol as substrates [41]. The sphingomyelinase activity of alpha-toxin also hydrolyzes SM to ceramide [14, 41–43] and this enzymatic activity is enhanced by DAG and other lipids that increase the negative curvature of the bilayer [43]. Genetically, engineered variants of alpha-toxin with point mutations at the active site exhibit a dramatic reduction in cytotoxic activity [17]. Therefore, the generation of DAG and ceramide in the plasma membrane is likely required for cytotoxicity. DAG and ceramide produced by alpha-toxin undergo spontaneous transbilayer translocation on model and erythrocyte membranes at rates modulated by membrane composition [44, 45]. Transbilayer motion rates decrease in the order DAG >> ceramide >> phospholipids [44]. DAG can freely diffuse through the membrane and change its physical properties, resulting in flip-flop that can occur in seconds to 1 min [30, 44, 46, 47]. On the other hand, the flip-flop motion of ceramide in the plasma membrane proceeds at a slower rate [48]. Ceramide can induce the transbilayer motion of other lipids and bilayer scrambling [48]. Thus, it has been suggested that the membrane dynamics induced by DAG and ceramide in cells treated with alpha-toxin is the key event in pathogenesis.

Endogenous PLC plays an important role in the pathogenesis induced by alpha-toxin [4, 22]. The treatment of A549 cells with U73122, an inhibitor of endogenous PLC, inhibited both toxin-induced clustering of GM1a and phosphorylation of TrkA at the marginal region of the plasma membrane. Furthermore, U73122 inhibited the toxin-induced release of IL-8 and the formation of DAG in A549 cells, but U73343, used as a negative control for U73122, did not. However, U73122 did not completely inhibit the events induced by alpha-toxin. Thus, activation of TrkA and endogenous PLC via phospholipid metabolism induced by the toxin itself plays an important role in the release of IL-8 by A549 cells. Alpha-toxin, but not an inactive variant, specifically induced phosphorylation of PLCγ-1 at the plasma membrane. Moreover, transfection of A549 cells with siRNA for PLCγ-1 inhibited GM1a clustering and IL-8 release induced by alpha-toxin, confirming the intermediate role of PLCγ-1 in the pathogenic activity of alpha-toxin in A549 cells. The pathogenic events induced by PLC from bacteria such as *P*. *aeruginosa*, *Bacillus cereus*, and *Clostridium botulinum* are likely caused by complex reactions involving phospholipid metabolism and protein phosphorylation, similar to that seen with alpha-toxin.

Monturiol-Gross et al. have reported that alpha-toxin binds to a receptor present in cholesterol-enriched membrane domains, becomes endocytosed with caveolin, and traffics through early and late endosomes and lysosomes, dependent on dynamin in DonQ cells [49]. Activation of PLCγ-1 in response to growth factors such as nerve growth factor, endothelial growth factor, and platelet-derived growth factor is required for motility and invasion (endocytosis) in different cell systems [50]. Therefore, activation of PLCγ-1 by alpha-toxin likely involves endocytosis of the toxin.

**Fig 8:**
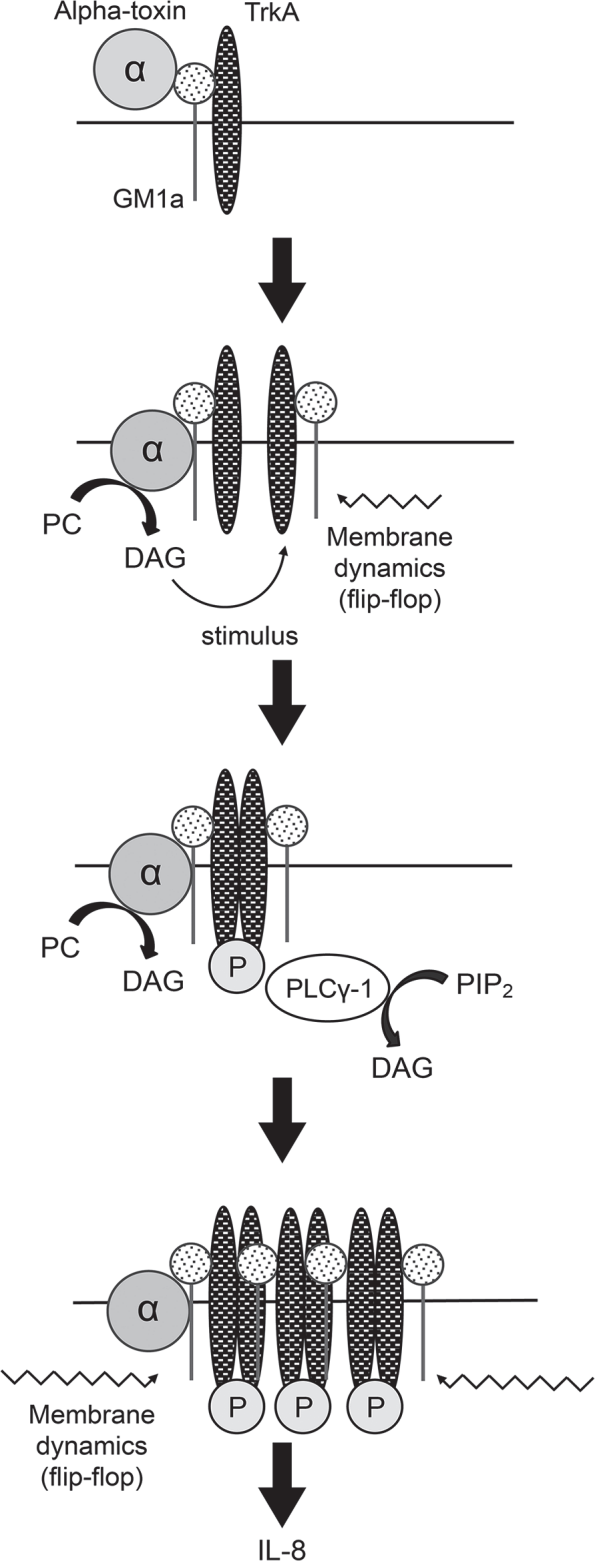
Schematic model of alpha-toxin-induced membrane dynamics and accumulation of the GM1a/TrkA complex

Based on our results, we propose the model detailed in Fig 8. Alpha-toxin specifically binds to GM1a and, through the enzymatic activity of alpha-toxin itself, induces DAG formation at the plasma membrane using PC as a substrate. The flip-flop motion of DAG influences membrane dynamics, promoting GM1a clustering and TrkA activation. TrkA activation leads to the phosphorylation of endogenous PLCγ-1, resulting in the increased formation of DAG. Finally, DAG formation leads to the increased activation of TrkA, triggering IL-8 release, which causes acute inflammation by recruiting and activating neutrophils. Thus, it appears that the control of phospholipid metabolism and phosphorylation in the cell membrane is an important target for the treatment of inflammation induced by the release of excessive IL-8 from cells treated with alpha-toxin.
